# Collective behavior and self-organization in neural rosette morphogenesis

**DOI:** 10.3389/fcell.2023.1134091

**Published:** 2023-08-10

**Authors:** Mattia Miotto, Maria Rosito, Matteo Paoluzzi, Valeria de Turris, Viola Folli, Marco Leonetti, Giancarlo Ruocco, Alessandro Rosa, Giorgio Gosti

**Affiliations:** ^1^ Center for Life Nano and Neuro Science, Istituto Italiano di Tecnologia, Rome, Italy; ^2^ Department of Physics, Sapienza University of Rome, Rome, Italy; ^3^ Department of Physiology and Pharmacology V. Erspamer, Sapienza University of Rome, Rome, Italy; ^4^ Departament de Física de la Matèria Condensada, Universitat de Barcelona, Barcelona, Spain; ^5^ D-TAILS srl, Rome, Italy; ^6^ Soft and Living Matter Laboratory, Institute of Nanotechnology, Consiglio Nazionale delle Ricerche, Rome, Italy; ^7^ Department of Biology and Biotechnologies Charles Darwin, Sapienza University of Rome, Rome, Italy

**Keywords:** neural tube, neural rosettes, complex system, morphogenesis, multi-agent system, collective behavior and dynamics, phase transitions

## Abstract

Neural rosettes develop from the self-organization of differentiating human pluripotent stem cells. This process mimics the emergence of the embryonic central nervous system primordium, i.e., the neural tube, whose formation is under close investigation as errors during such process result in severe diseases like spina bifida and anencephaly. While neural tube formation is recognized as an example of self-organization, we still do not understand the fundamental mechanisms guiding the process. Here, we discuss the different theoretical frameworks that have been proposed to explain self-organization in morphogenesis. We show that an explanation based exclusively on stem cell differentiation cannot describe the emergence of spatial organization, and an explanation based on patterning models cannot explain how different groups of cells can collectively migrate and produce the mechanical transformations required to generate the neural tube. We conclude that neural rosette development is a relevant experimental 2D *in-vitro* model of morphogenesis because it is a multi-scale self-organization process that involves both cell differentiation and tissue development. Ultimately, to understand rosette formation, we first need to fully understand the complex interplay between growth, migration, cytoarchitecture organization, and cell type evolution.

## 1 Introduction

Stem cell biology and developmental biology can be regarded as two sides of the same process. The morphogenesis of living tissue can be studied either at the cell differentiation level or at the organ development level, in analogy to what happens in physics, where matter can be observed either at the microscopic scale of the atoms or at the macroscopic scale we experience in everyday life. To understand the relation between the atoms scale and the material scale, physics has developed a multi-scale approach based on statistical mechanics (Anderson, 1972). Here, we aim at discussing how such an approach can be used to understand the relationship between cell and tissue scales, in the special case of neural rosette formation.

**FIGURE 1:**
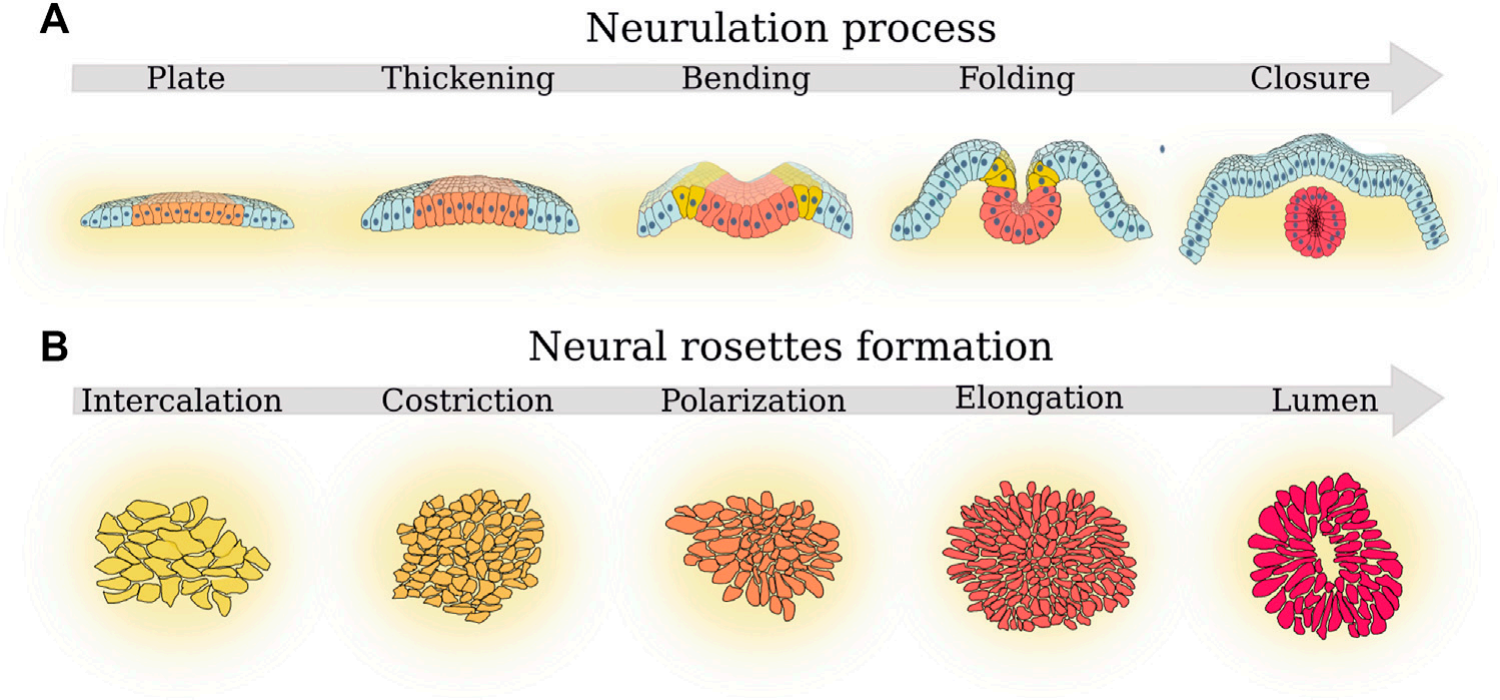
Parallel between the principal phases of neurulation and rosette formation. **(A)** The five phases of neurulation that determine neural tube morphogenesis (Karzbrun et al., 2021): 1) plate formation, a plate of cells from the ectoderm (light blue cells) became neural ectoderm cells (orange cells); next, 2) in the thickening phase, such neural plate thickens and then start 3) bending. Cells at the border of the neural plate further differentiate becoming neural crest progenitors (yellow cells). In a subsequent 4) folding phase, the margins of the neural plate come together. Finally, 5) in the closure phase, the neural plate completes the folding process and forms the neural tube (red cells). Progenitor crest cells migrate toward other regions of the embryo. **(B)** The five fundamental cell morphological changes described by Hříbková et al. (2018) during rosette formation. First, 1) cells form confluent layers (cell intercalation) and then 2) such cells tend to form a thicker and more dense layer (cell constriction). Next, 3) cells start forming defects (cell polarization). In the next phase, 4) more cells join the defect (cell elongation) until 5) a lumen is formed in the place of the defect (lumen formation).

The term “neural rosette” indicates the arrangement of elongated neuroepithelial cells in a blossom-like fashion, typically observed during *in vitro* differentiation of pluripotent stem cells (Okabe et al., 1996; Zhang et al., 2001). In particular, the architecture of the neural rosette recapitulates fundamental aspects of the development of the neural tube, which constitutes the central nervous system (CNS) primordium. The latter can be thought of as a system composed of different tissue types organized in a very specific order and found to be practically identical in all healthy individuals. In brief, the neural tube emerges from the neural ectoderm layer which folds itself and generates this extremely symmetric cylindrical structure (Detrait et al., 2005). This morphogenetic process evolves through a series of stages (see Figure 1A). Each stage in the evolution of the spinal cord is characterized by a very specific architectonic structure and cell differentiation program, where minor deviations from these fundamental structures may result in developmental disorders. Despite many efforts have been spent to model both aspects of such process (Townes and Holtfreter, 1955; Antoine et al., 2021; Karzbrun et al., 2021; Moon and Xiong, 2022), at the current stage, both stem cell models of differentiation and tissue patterning models failed to completely explain the mechanics involved in this complex process because they exclusively focus respectively on cell differentiation or patterning. In fact, while from a molecular perspective, cell differentiation is determined by the combination of stochastic events, cell signaling, and environmental cues that can be understood at the single-cell level without considering the complex interactions typical of collective behavior (Yamanaka, 2009); recent discoveries have shown how differentiating pluripotent stem cells have the potential to self-organize in functional cortical (Eiraku et al., 2008), retinal (Nakano et al., 2012), cerebral (Lancaster et al., 2013) and intestinal organoids (Watson et al., 2014), thus demonstrating a link between tissue architecture and mechanical interaction with cell differentiation. Notably, these processes do not seem to be coordinated by specific leader cells, that are trivially recognizable but appear to be guided simultaneously by signaling gradients and self-organization that together guide cell differentiation at the single-cell level.

Generally, we broadly understand how a cell executes the program encoded in the genetic code, as a form of computer code or a cooking recipe. In this perspective, proteins are generated in response to external stimuli which can be chemical or mechanical according to the information encoded in the gene co-activation network. Thus, self-organization in tissue development could be thought of as a distributed optimization problem, in which the final cytoarchitecture is the desired optimal solution (Hassan and Jeff, 2020). Unfortunately, until the work on ant colony optimization by Dorigo and Birattari (2011) showed how leaderless algorithms inspired by nature can be effective in solving real-world optimization tasks, distributed leaderless systems without a master processor were thought to be unable to converge. In this respect, Dijkstra (Dijkstra, 1974) proved that any deterministic distributed algorithm, without a master processor, is not guaranteed to solve a distributed optimization problem in a finite time. In practice, this work was loosely interpreted to mean that a leaderless morphological event could not always be able to complete itself in the desired final state. Nevertheless, (Bistarelli and Gosti, 2010; Gosti et al., 2011; Gosti, 2013) showed artificial stochastic multi-agent systems able to find the solution to collective problems and are guaranteed to find a solution given sufficient time with a probability equal to one. This allows us to speculate that neural rosette morphogenesis may be described as a collective behavior process that is decoded in the cell’s gene co-expression network as a stochastic distribution program.

In this framework, cell differentiation may be regarded as a form of collective behavior similar to the behavior we find in condensed matter physics, where different states of matter are linked with each other through phase transitions (Jeffrey, 2022). To better comprehend this analogy and understand how complex matter physics can help us understand rosette formation, we can consider, for example, the magnetization of a ferromagnetic material, where the magnetic dipoles of the atoms on a lattice interact through pairwise interactions, and their states are perturbed by thermal noise. These pairwise interactions are usually modeled into an energy function that is minimized when the magnetic dipoles are aligned. Above a certain critical temperature, called Curie temperature, the system is in a disordered state and the magnetic dipoles are random. When the temperature is lowered and the system gets close to the critical value, the information starts propagating and the phase transition starts. As the temperature continues to lower and the phase transition ends, the magnetic dipoles in each domain align in a certain direction that is collectively determined. When magnets are aligned the energy function is at its minimum. This process is known as symmetry breaking since the ferromagnetic material loses its initial stochastic symmetry, due to the initial random orientation of its magnetic dipoles, and chooses a direction of orientation. To understand symmetry breaking in ferromagnetic materials, physicists developed mathematical methods to describe collective decision-making and behavior. Subsequently, several studies have exploited the analogy with ferromagnetic systems to model collective behavior in complex systems (Vicsek and Zafeiris, 2012). The biological systems that were modeled in this collective behavior framework are several, ranging from flocks of birds (Ballerini et al., 2008) and insect swarms (Attanasi et al., 2014), to worms’ (Sugi et al., 2019) and cells’ (Szabó et al., 2006) populations. These studies usually formulate an effective energy function that presents as optimal states the kind of collective behavior experimentally observed. Thus, given this effective energy function, the collective behavior becomes an emergent property that is the result of an energy function minimization. To sum up, if we could model the development of the different phases of neural rosette formation as a sequence of collective behavior processes, this would imply that the transition to progressive stages of tissue development could be modeled as a sequence of transitions between couples of less-orderly/less-differentiated stages and more-orderly/more-differentiated stage.

In this light, we believe that rosette formation can be a natural benchmark for studying self-organizing in morphogenesis. In fact, first of all, it is a simpler 2D model of neural tube formation, which we can use to later understand and develop a more complex 3D mathematical model of neural tube formation. Secondly, it is not trivially reduced to previous conceptual theoretical frameworks. More specifically, the advantage of studying 2D neural rosette formation as opposed to 3D neural tube formation is that neural rosettes experiments are much more feasible from a biological and computational point of view allowing us to repeat several experiments in order to analyze its sensibility to initial conditions and different control parameters. This allows us to scale up the number of model parameters and conditions that we can test. Contrarily, 3D computational models require extensive computational time even for single simulation runs (Chen and Brodland, 2008) and 3D biological *in vitro* models are practically impossible to replicate several times (Karzbrun et al., 2021). Overall, the objective of this review will not be to cover the literature on neural tube morphogenesis, but to review the interdisciplinary literature related to rosette morphogenesis which discusses the complex mechanical and chemical processes guiding self-organization and collective behavior in rosette morphogenesis and to shows how this paradigm can be used to better understand neural tube development.

Indeed, we will elaborate on these aspects, discussing neural rosette formation from a statistical physics point of view. In particular, we will start briefly presenting the different schools of thoughts that emerged in the contest of morphogenesis modeling (see Section 1.1); then we will recapitulate the key elements of rosette formation and their link with the neurulation process (see Section 1.2) and we introduce the concept of a complex system, explaining why rosette are an example of a complex process in which cells manifest a collective behavior (Section 1.3). Then, e will focus on the various approaches that have been adopted to study such systems. We will deal with the Stem Cell perspective (Section 2.1), according to which, cells undergo a progressive cascade of differentiation events leading to the terminal cells. In Section 2.2, we will instead face the problem of modeling the spatial organization the cells assume in the rosette formation, describing the most famous mathematical models derived to address this issue. Then, in Section 2.3, we will discuss the Dynamical Cell perspective: an understanding of morphogenesis based solely on cell potency and lineage will tend to avoid the fact that cells themselves are dynamical systems that grow, divide, and proliferate. Fundamental characteristics that any model of neural tube development should be able to explain from a macroscopic tissue-level perspective are homeostasis and organogenesis. In Section 3.1, we will introduce the concept of phase transition and the possible link with neural rosette formation. Finally, we will discuss what is still missing (Section 4.1) and what may help making progresses in the field (Section 4.2), speculating on the possibility to view morphogenesis as a phase transition process similar to what happens in many other physical systems (Section 4.3).

### 1.1 Overview on the morphogenesis modeling

While the recent availability of quantitative experimental techniques and increased computational power is bursting the studies on morphogenesis, first speculations on the processes behind such phenomenon date back to Aristotle, who introduced -on a phylosophical level-the epigenetic hypothesis, postulating that structures develop gradually. This idea was in contrast with the prevailing preformationism point of view in which structure did not emerge but development corresponded just to change in size [see (Mayr, 1998) for a comprehensive discussion]. A significant shift towards a mechanical and geometrical understanding of morphogenesis occurred with Galileo Galilei, who observed that organisms change their shape in response to the loads they bear (White and Kearney, 2014).

Followed in the late 19th century, by the works of His and Roux, who played a pivotal role in shifting the focus of developmental biology from evolution to understanding the underlying mechanisms of development, exploring two aspects of development: self-differentiation and dependent-differentiation, conducting experiments on embryos (Richardson and Keuck, 2022). The 20th century witnessed significant discoveries on the relationship between physical forces and shape generation, under the influence of D’Arcy Thompson. In fact, while other contemporary scientists focused on experimental analysis, D’Arcy Thompson’s investigations were based on a mathematical approach and the idea that morphogenesis was based on physical forces in shaping organisms (Wentworth D’Arcy Thompson, 1917). Although his observations and calculations were purely hypothetical, they laid the foundation for further scientific advancements. A decade later, Alan Turing proposed the reaction-diffusion mechanism (Turing, 1952), able to explain pattern formation through the interaction of fast-diffusing inhibitors and slow-diffusing activators, resulting in periodic patterning through diffusion instability. Turing’s work demonstrated the feasibility of representing morphogenetic patterns using simple mathematical rules. However, it took almost five decades for the proposed mechanism to gain significant traction in the field.

Lewis Wolpert’s conceptual framework of positional information in morphogenesis was another important milestone. Inspired by Hans Driesch’s observations in sea urchin morphogenesis, positional information proposed that a cell determines its fate based on its position relative to other parts of the organism. This position is characterized by the concentration of a morphogen, which the cell interprets to make fate decisions (Vargesson, 2019). The advent of digital computers greatly accelerated research on morphogenesis by enabling computational modeling. The reaction diffusion mechanism gained significant interest and was successfully tested using advanced mathematical models and improved experimental techniques. Additionally, newer approaches consider mechanical, electrical, and environmental cues in understanding morphogenesis. However, these factors are often investigated independently, highlighting the need for a more integrated approach that considers the multiscale nature of morphogenesis and the synergistic effects of diverse signals. Agent-based models have emerged as an alternative for modeling morphogenesis in complex and heterogeneous environments. Such models use autonomous agents that represent various entities, such as molecules, cells, or organisms. These agents have defined behaviors and interact with each other and their simulated environment based on predefined rules. The dynamics of an agent can encompass a range of actions, and decision-making is often probability-based (Brodland et al., 2010; Nielsen et al., 2020). A more detailed description of such different approaches will be given in the following sections.

### 1.2 Neural rosette as a model for neurulation

Rosettes are multi-cellular structures characterized by a radial symmetry that resembles a rose or a blossom (Wippold and Perry, 2006). The cells that form such a structure present an inhomogeneous spatial organization such that the adhesion molecules and junctions that hold the cells together are localized at the center of the rosette where they form the rosette lumen; this is referred to as apical-basal polarity (Harding et al., 2014). Usually, rosettes are transient structures that in certain cases recapitulate the form of the adult organ. They tend to develop from irregularities in the disposition of cells embedded in the regular patterns formed by epithelial sheets. In this irregular disposition, a larger than the average number of cells adhere to each other. These irregular local patterns resemble topological defects in condensed matter (Wenzel et al., 2019). Rosettes are observed during the formation of diverse organ systems (Harding et al., 2014), such as the zebrafish lateral line primordium (Ma and Raible, 2009), the vertebrate pancreas (Alethia VillasenorChong et al., 2010), the *Drosophila* epithelium (Todd Blankenship et al., 2006), and in the adult neural stem cell niche (Mirzadeh et al., 2008). Furthermore, under the proper culture conditions, both human embryonic stem cells (hESCs) and induced pluripotent stem cells (hiPSCs) undergo morphogenic changes and form neural rosettes, similar to the ones described in *in vivo* systems (Zhang et al., 2001; Elkabetz et al., 2008; Chen et al., 2012; Karus et al., 2014). In particular, neural rosette show accumulation of apical ZO-1 and N-cadherin at their center, which then corresponds to where the lumen is formed (Elkabetz et al., 2008) (ZO-1 and N-cadherin are respectively a tight junction-associated protein and a transmembrane protein that mediates cell–cell adhesion). Furthermore, cells in neural rosette show nuclear expression of PAX6, a “master control” gene for the development of different tissues, activating when the cells start to differentiate in the cells specific to that particular tissue. In neural rosette cultures, it is considered a marker of primitive neural ectoderm cells because it is been shown that PAX6-positive cells can differentiate into any region-specific neural progenitor (Elkabetz et al., 2008; Zhang X et al., 2010). Consequently, the cells constituting neural rosette are considered neural progenitor cells.

All the aforementioned features make neural rosettes a widely used model for studying *in vitro* the development of the neural tube [even though, current research points out how neural rosette formation presents certain specific affinities peculiar to secondary neurulation as opposed to the primary one (Fedorova et al., 2019)]. *In vivo*, the morphogenesis of the neural tube, also called neurulation, starts with the emergence of the neural plate which folds itself into a singular neuroepithelial tube that spans the entire rostro/caudal axis of the embryo’s dorsal plane. The neural tube serves as the primordium of all central nervous system tissues. Furthermore, this cylindrical polarized structure of cells becomes the center for the spatial organization of the cells forming the spinal cord (Knight et al., 2018). During neural tube cytoarchitecture development, Interkinetic nuclear migration (INM) takes place. In this process, first observed by Sauer (1935), nuclei migrate between the apical and basal ends of these cells, allowing for the proliferation of neural progenitors at the tube’s apical surface while daughter cells migrate radially towards the basal surface to complete differentiation (Gilbert, 2000; Lui et al., 2011). It is interesting to note that INM is observed also in neural rosettes (Ziv et al., 2015). *In vivo*, different conditions can cause the incorrect development of the neural tube, which can either be completely eliminated, fail correct neural folding, or develop into more than one tube. For example, N-cadherin disruption destabilizes the polarized cytoarchitecture inhibiting the development of the central nervous system (Zhang J et al., 2010). Alternatively, the formation of multiple neural tubes during development causes congenital abnormalities such as dipolmyelia and diastematomyelia (Cogliatti, 1986; Testoni et al., 2010). Failures in neural folding are among the most common birth defects, affecting around 1 in 1,000 pregnancies (Wallingford et al., 2013; Lee and Gleeson, 2020). Therefore, understanding the mechanisms that guide the self-organization of a single neural epithelium tube with either an hESC or an hiPSC organotypic tissue has fundamental implications (Lee et al., 2017; Knight et al., 2018).

Neural tube morphogenesis follows five phases of neurulation: neural plate formation, thickening, bending, folding, and closure (Figure 1A). In the neural plate formation phase, a portion of the cells of the ectoderm (the outer layer of cells in the developing embryo) become neural ectoderm cells. In the thickening phase, the neural plate thickens as the neural ectoderm cells begin to differentiate and grow. In the bending phase, the neural plate starts to curve and shape into a tube-like structure. In the folding stage, the margins of the neural plate come together and begin to fold inward. Finally, in the closure phase, the neural plate completes the folding process and fully closes to form the neural tube. The study of Karzbrun et al. (2021) presented an *in vitro* experimental 3D model that recapitulates the self-organization of neural tube morphogenesis with human iPS cells. This neural tube morphogenesis model allows us to directly observe the five phases of neurulation, which is otherwise only observed in non-primate models (Karzbrun et al., 2021). This model shows that single-cell proliferation potential is not sufficient to determine the outcome of neural tube formation, but that the control of the initial size, cell density, and shape of 2D stem-cell cultures and neighboring cells is fundamental as well. Karzbrun et al. (2021) study proves that it is not just the potency of the cell culture that determines self-organized morphogenesis, but also other collective behavior properties must be considered. Thus, neural tube folding requires the production of mechanical forces and the regulation of tissue mechanical properties. Furthermore, it must interact with the neighboring tissues and the environment: the extracellular matrix (ECM) (Rifes and Thorsteinsdóttir, 2012; Zhu et al., 2015; Guillon et al., 2020; Molè et al., 2020), the extraembryonic substrates (Münster et al., 2019), and the lumen (Dumortier et al., 2019). In this context, the development of mechanical models is fundamental to understanding how morphogenesis allows the self-organization of cells in tissues and organs (Moon and Xiong, 2022).

Neural rosettes are an excellent model to study neural tube morphogenesis because they reproduce the five fundamental features of neural tubes: 1) radial structure, 2) an evident lumen structure, 3) gene expression profile (including the characteristic transcription factor PAX6 and the membrane junction N-cadherin), 4) cytoarchitecture dynamics, 5) potential to develop several cell lines. Consequently, neural rosettes mimic the development of a single section of the neural tube perfectly, and for this reason serve as a 2D model of the 3D neural tube formation process. Furthermore, Hříbková et al., 2018 identified five fundamental cell morphological changes (Figure 1B): cell intercalation, cell constriction, cell polarization, cell elongation, and lumen formation. In the cell intercalation stage, cells form confluent layers of neighboring cells adherent to each other. In the constriction stage, cells tend to form a thicker and dense layer. In the polarization stage, cells tend to form membrane junctions, which are unevenly localized around specific points in the cell layer where more cells join each other. These centers became defects or in other words points in the cell layer where the cells form an unevenly dense pattern compared to the rest of the evenly organized cell layer. In the elongation stage, more cells join the defect. In the lumen formation stage, a lumen is formed in the place of the defect. With a certain degree of approximation, these five cell morphological changes recapitulate the five stages of neurulation, even if they are the result of a state-of-the-art synthesis and have not yet been substantiated by the statistical analysis of specific morphological markers on biological models. Nevertheless, they evince the role of self-organization and collective behavior because they show how the cells need to coordinate their movement and shape through deformation and stiffness to form a rosette, while at the same time determining each cell’s differentiation state. Indeed, the cells inside the rosette are PAX6 positive, while the cells outside the rosette do not always differentiate.

### 1.3 Complex systems and collective behavior

A complex system is characterized by many individual members that are connected by relations, and/or interactions. Examples of complex systems go from human social networks to ant colonies, from the fractal shape of bronchi and bronchioles in the lung to the organization of the central and peripheral neural systems. These systems can be described using networks, in which the links that connect nodes depict relations and interactions. More importantly, what characterizes a complex system is that it cannot be fully described by the characterization of individual parts separately, but it must be studied as a whole. This is because the response of every single component is characterized by its state and the influence of the other parts connected to it (Rutter et al., 2017). Such concept is at the basis of the complexity theory.

If we want to study a complex system in a classical experimental framework, we would require to assay all the responses for each treatment and the configuration of all the components of the system. Consequently, the experimenter would ideally have to control all the relations and interactions between the parts. For this reason, in cell biology, to avoid this problem, the experimenter often assumes that the system behaves as a homogeneous container of single parts, and studies the entire system as an idealized collection of these single parts (Barabási et al., 2011; Dimitrakopoulou et al., 2014). For example, neural tube formation may be simplified and explained exclusively as the differentiation of the neuroectoderm cells that form the neural plate. But this approach would disregard the mechanical interactions that are required for the neural plate to fold in a neural tube. This implies as well that we would disregard the role that mechanical interactions and chemical signaling have on single-cell genetic expression, and we would have to reintroduce this in the model. Indeed, as discussed in the previous section, to develop the neural tube, the ectoderm cells must collectively coordinate their transcriptomic state change to become neural-ectoderm cells. Furthermore, these differentiating cells mechanically change their surrounding environment to organize themselves spatially in the neural tube.

Neural rosettes are a good example of a complex system because they can be easily represented as a network in which the nodes are cells, and the edges represent the pairwise interactions between cells that are either mechanical or chemical. Mechanical interactions are mediated through the elastic/dissipative interactions between each other and the substrate that are the result of the cell’s spacial organization and cytoarchitecture. Chemical interactions are established by (but not limited to) soluble proteins that may either diffuse in the media or be allowed by membrane junctions connecting pairs of cells. In rosettes, it is observed that only the cells in the rosette and in the large clusters express PAX6, while the other cell in the same culture with the same differentiation factors do not express PAX6. This points to the fact that cell differentiation is not only guided by molecular differentiation factors but is as well the result of mechanical forces, and direct molecular pairwise interaction (Hříbková et al., 2018) that determines an emergent collective behavior.

## 2 Current perspectives

Here, we will discuss more in details the different perspectives from which morphogenesis can be addressed from a statistical physics point of view. In particular, we will describe stem cell differentiation, and the models developed to study tissue organization. Finally, we will discuss models that represent cells as dynamical systems subject to biological noise.

### 2.1 The stem cell scale perspective

**FIGURE 2:**
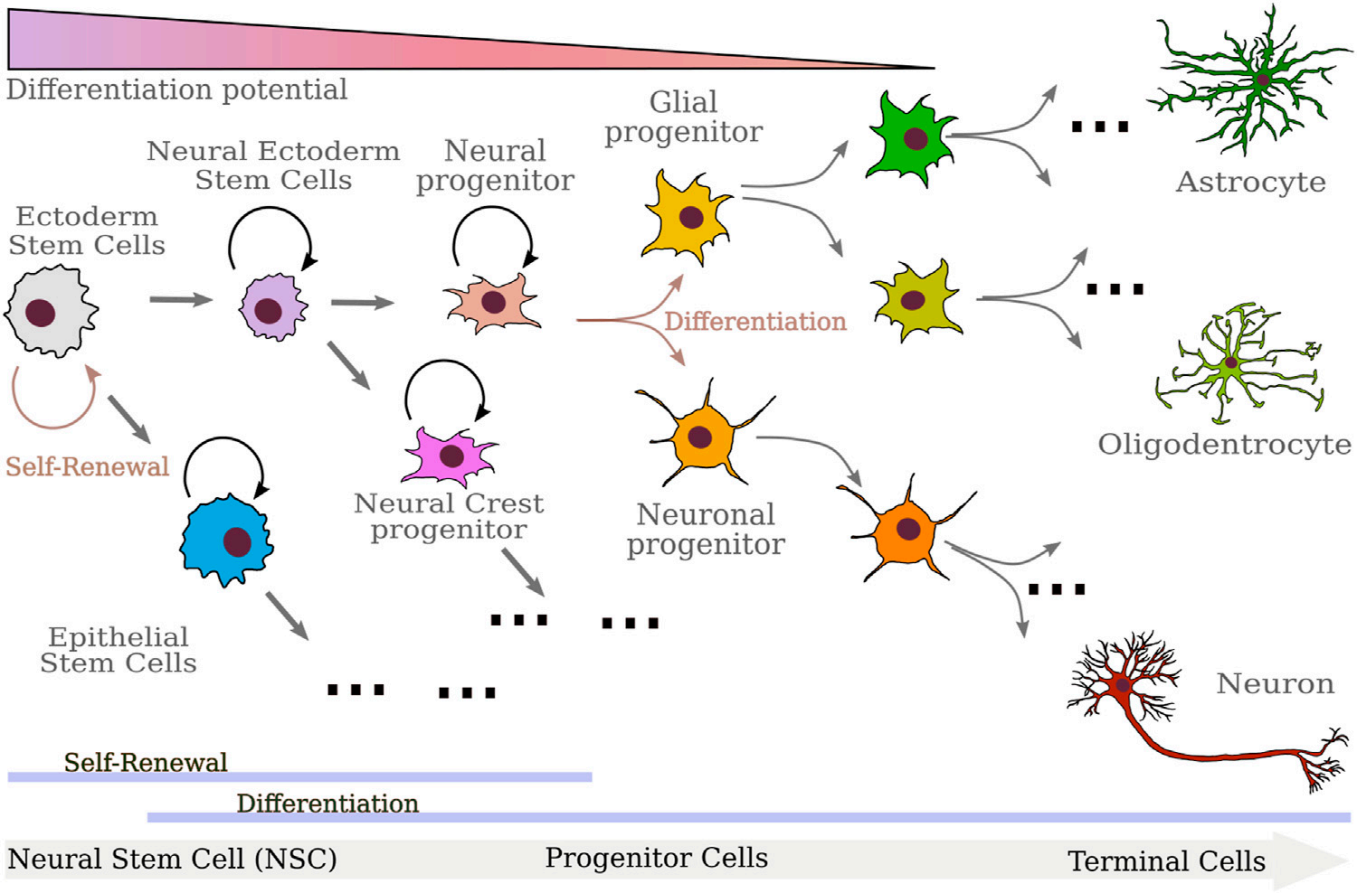
Sketch of the differentiation progression of the cells involved in neurulation. Starting from Ectoderm Stem cells, neural ectoderm and epithelial stem cells emerge. Neural ectoderm stem cells further differentiate in the progenitors of neural and neural crest cells. While neural crest cells will in turn differentiate into several different mature cells, neural progenitors end up producing astrocytes, oligodendrocytes, and neurons. As differentiation proceeds, cells progressively lose differentiation potential and renewal ability.

Stem cell models of differentiation and tissue patterning try to explain development by studying the same process at two different scales. Stem cell models focus on the changes at the cell level and tissue patterning models focus on the changes that are present at the tissue level. At the cell level, stem cells are the fundamental progenitor cells that generate all the cells that form the adult organs, and can self-replicate. When a tissue is injured, stem cells produce cells apt to form the new tissue. Each different type of stem cell is characterized by the organs or tissues their progeny are going to form, e.g., skeletal muscle stem cells produce skeletal muscle. Each stem cell is associated with a lineage tree formed of all the cell types that are part of its progeny *in vivo* (see Figure 2). The branches that form the stem cell lineage select different cell fates and produce the different types of cells that form the adult organ. In this picture, development is a one-way process in which cells gradually lose the potential to generate different types of cells and become more specialized cells. In general, stem cells have to balance the tasks of generating new cells and maintaining genetic integrity, because excessive cell replication may lead to a greater probability of DNA replication errors. To achieve this aim, stem cells proliferate slowly and generate new stem cells and progenitor cells. The latter proliferate frequently, and generate further progenitor cells and terminal cells. These progenitor cells have the fundamental role of expanding the size of the stem cell progeny, thus allowing stem cells to be in a relatively small number compared to terminal cells. Since progenitor cells divide more often than stem cells, stem cells can be protected in stem cell niches, and do not risk accumulating dangerous mutations caused by frequent divisions. Terminal cells are fully differentiated cells that usually characterize the tissue and perform specific functions. Thus, they cease to divide. In this framework, we expect cells to become more specific as they differentiate and do not change their specialization as it is acquired: we would expect neural ectoderm cells to differentiate in respectively neural tube cells and neural crest cells and that neural tube cells do not change their fate to neural crest cells or revert to neural ectoderm cells. The gradual loss of potency is what is observed in most cases with some important exceptions that show the limits of this framework.

In fact, Gurdon’s experiments on *Xenopus laevis* (Gurdon et al., 1958), the birth of cloned sheep (Wilmut et al., 1997), and finally induced pluripotent stem cells (iPSCs) (Takahashi and Yamanaka, 2006) prove that cells can be de-differentiated artificially and brought back to a pluripotent state. Furthermore, current research data on cell reprogramming and trans-differentiation is challenging the concept that cell fate determination is a deterministic unchangeable choice. In particular, (Antoine et al., 2021) discusses how neural crest cells appear to undergo a natural *in vivo* reprogramming event that allows their progeny to differentiate into a lineage with characteristics analogous to epiblast stem cells. Indeed, the reprogramming of the neural crest lineage is responsible for the formation of the majority of facial mesenchyme (Antoine et al., 2021). Nevertheless, the concept of gradual loss of potency in differentiation is still the general conceptual framework (Yamanaka, 2009). The stem cell lineage perspective has been very successful in explaining systems like the blood and skin for two different reasons respectively. In the first case, blood is an amorphous liquid, thus it does not have a proper spatial organization. Meanwhile, in the second case, skin is specially organized only in depth (i.e., going from epiderm to derm) and it is basically homogeneous in the transverse dimensions. Indeed, skin organization along the depth dimension is the result of its cells’ differentiation history. The stem cells form the deepest layer (the basal layer) and the terminal cells are at the more external layer with all the progenitors in the central layers, which constantly replace the terminal cells that are shed.

Taking these observations into consideration, it is important to point out that the stem cell lineage perspective forgets the role of spatial organization, size, single-cell transcription errors, partitioning errors, and chemical/mechanical signals. Consequently, without the introduction of stochasticity and mechanical and chemical signaling feedback between the different tissue and cell types, it is difficult to conceptualize how a cell can be pushed to re-program itself. For these reasons, the stem cell lineage perspective does not allow us to fully understand the relationship between cell differentiation and tissue spatial organization in the spinal cord, and other organs characterized by the spatial organization of different cell types.

### 2.2 The spatial organization perspective

**FIGURE 3:**
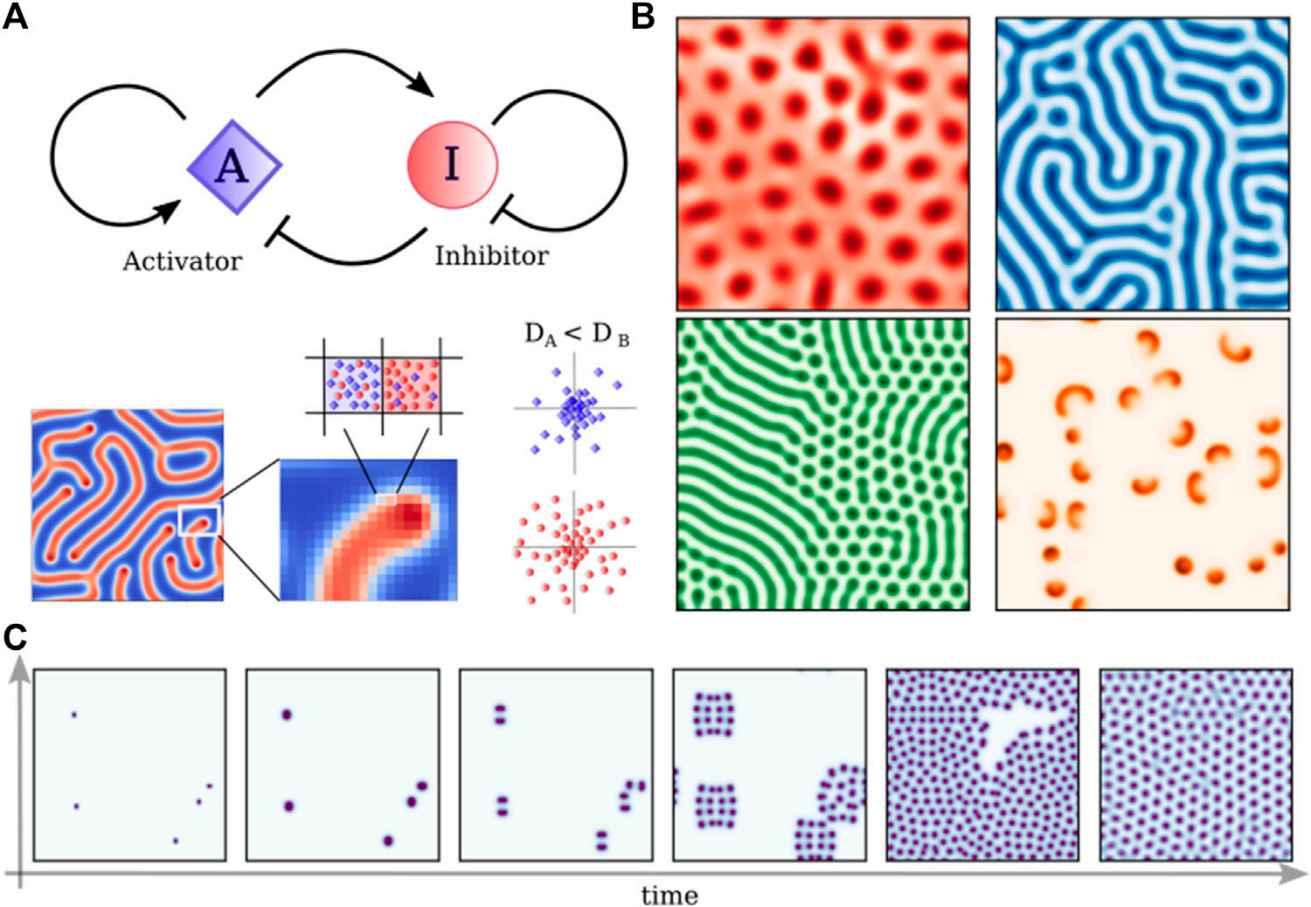
Turing reaction-diffusion models. **(A)** Schematic representation of a simple Activator-Inhibitor circuit, which can be represented by the set of Eq. 1 with *R*_*A*_ = *I* ⋅ *A* ^2^ − (*f* + *k*)*A* and *R*_*I*_ = −*I* ⋅ *A* ^2^ + *f*(1− *I*). Differences in the diffusion of the two competing molecules create patterns that depend on the specific set of parameters. **(B)** Patters obtained solving Eq. 1 with different sets of parameters: f = 0.02430, k = 0.05174; f = 0.028, k = 0.056; f = 0.03, k = 0.06; f = 0.00713, k = 0.04651; **(C)** Time evolution of the “cell-dividing” pattern (f = 0.028, k = 0.062) starting from five random seeds.

Looking at morphogenesis from the point of view of tissues, one observes tissue cells undergoing spatial reorganizations that give rise to the formation of patterns. Numerous theories have been put forward with the goal to unravel the mechanism by which initially equivalent cells in a developing tissue assume complex forms and functions, i.e., patterns [for dedicated reviews see, for example, (Roth, 2011; Landge et al., 2020)]. Few of them are as universal as the reaction-diffusion (RD) model proposed in 1952 by Alan Turing (Turing, 1952). His model assumes that cells are capable of sensing and producing morphogens and that their position in space is fixed. In particular, the spatio-temporal dynamics of two morphogens is described by:

$$\frac{dA}{dt}=D_{A}\nabla^{2}A+R_{A}\left(A,I\right)\text{and}\;\frac{dI}{dt}=D_{I}\nabla^{2}I+R_{I}\left(A,I\right)$$ (1)

where *A*(x, *t*) and *I*(x, *t*) represent the concentrations of the two morphogens, *D*_*A*_ and *D*_*I*_ the diffusion coefficients, and *R*_*A*_ (resp. *R*_*I*_) is a function describing the reaction that takes place between species A and I (see the sketch in Figure 3A).

The simplest and most well-known example of an RD system is the activator-inhibitor system (Gierer and Meinhardt, 1972), consisting of two diffusible components that interact through specific reactions. Turing’s two-component system has linear reaction terms, while successive works, like that of Meinhardt and Gierer (Gierer and Meinhardt, 1972), proposed a model with biochemically more realistic nonlinear reaction: $R_{A}=(\frac{A^{2}}{\left(1+k_{A}A^{2}\right)}+A_{0})/I+\mu_{A}A$ and *R*_*I*_ = *A* ^2^ − *μ*_*I*_ *I*. In this model, activator A promotes its own synthesis and that of inhibitor I, which in turn inhibits the activator and itself. Interestingly, this system can self-organize from an initially uniform distribution to give rise to stationary periodic patterns of activator and inhibitor concentrations. Such patterns with a characteristic periodicity or wavelength are termed Turing patterns.

Another example is given by the Gray-Scott choice for the reaction terms. In fact, setting *R*_*A*_ = *I* ⋅ *A* ^2^ − (*f* + *k*)*A* and *R*_*I*_ = −*I* ⋅ *A* ^2^ + *f*(1 − *I*), the Gray-Scott system models a chemical reaction of the form: *I* + 2*A* → 3*A*, where the morphogen I is consumed, while A is produced. To maintain the reaction, a feeding rate and killing rate are introduced with the parameters *f* and *k*, respectively. Note that the removal of *I* can also be described by another chemical reaction that turns *I* into an inert product via the rate *k*.

Despite the specific form of the reaction terms, the RD model shows how given two different morphogens we can obtain standing waves forming a stable periodic pattern (see Figures 3B, C for some examples), having peculiar properties. In particular, such a system is able to present truly self-organized patterns in the absence of initial asymmetries, thereby generating positional information. Moreover, many different patterns are formed by simply varying the reaction and diffusion parameters (see Figures 3B, C), which could in principle account for the structural and morphogenetic diversity of life forms. Finally, RD patterns are responsive to external perturbations and can regenerate after perturbations (Landge et al., 2020). Fundamental improvements in genetic engineering and computer science showed how this simple paradigm could reproduce some patterns observed in nature as in the case of leopard-coat or sea shell (Kondo and Miura, 2010), however, it remains unclear how commonly the principles of RD systems are actually realized in living systems. Moreover, tissue patterning disregards the importance of cell migration and differentiation. In particular, the massive collective cell migration that allows the neural tube to fold itself is completely unexplained.

Meanwhile, the early mechanical sorting experiments done by Townes and Holtfreter (1955) shows how not only chemical signals but also mechanical interaction can produce patterning in live tissue. In Townes and Holtfreter’s cell sorting experiments, cells dissociated by amphibian embryos were mixed in a homogeneous aggregate. If these cells are put in culture, they can autonomously rearrange themselves in structural arrangements that mirror the *in vivo* organization. Krens and Heisenberg (2011) explains how this sorting process is analogous to how immiscible liquids develop a surface tension and sort themselves in distinct regions. Townes and Holtfreter proposed that mechanical cell sorting can explain the folding of the neural ectoderm in neural tube formation. Unfortunately, its limit is that this model is unable to explain cell differentiation. It only explains sorting once the cells are already differentiated, and the mechanical forces required for the important cell dislocations in space. Both tissue patterning and mechanical cell sorting have a role in spinal cord development, but by themselves, they are unable to describe the mechanics behind this process.

### 2.3 The cell as a dynamical system (plus noise) perspective

As we discussed in Section 2.1, an understanding of morphogenesis based solely on cell potency and lineage will tend to avoid the fact that cells themselves are dynamic systems that grow, divide, and proliferate. Fundamental characteristics that any model of neural tube development should be able to explain from a macroscopic tissue-level perspective are homeostasis and organogenesis. Homeostasis is the ability of an organ to keep its size and shape even after perturbations, while organogenesis is the process of the development of tissue with a distinctive spatial configuration. Contrary to what we observe in a homeostatic tissue, in a generic steady-state culture configuration in which the cells’ nutrition is constant and cell mortality is low, homogeneous cell populations grow exponentially as each cell doubles with given doubling times (Van Heerden et al., 2017; Enrico Bena et al., 2021). La Porta et al. (2012) showed how Markovian branching models can be used to model the growth of such populations. Given a certain stem cell and its differentiation lineage, a Markovian branching model exhibits three different steady-state configurations (La Porta et al., 2012). Under certain conditions, we get linear growth or exponential growth, and under the proper balance between the death rate and division rate, it gives rise to a population that grows up to a fixed size. However, those state are not a stable under perturbations and the model does not capture homeostasis since it is unable to correct the population size after cell death or damage, i.e., if the cell population suffers a wound and loses an important part of its stem cell population it cannot regenerate the lost population. These observations imply that cells must have a mechanism that allows them to collectively tune their growth rate and their death rate. Hannezo et al. (2014) show how contact inhibition and mechanical stresses in the epithelial sheet influence tissue homeostasis. These considerations point out how an explanation of tissue development based only on stem cell differentiation omits a description of how cells organize themselves mechanically and in space.

Furthermore, without going into the specifics of the cell cycle, we usually assume that there is an initial mother cell that divides into two daughter cells thus it must duplicate its genetic material and cellular components. A mother cell may divide its internal components either symmetrically, or asymmetrically depending on respectively if the daughters are identical to the mother, or if the two daughter cells differ in size, cellular components, and/or differentiation potential. The way cells divide their components depends on the particular types of the cells’ components. In general, there are three cases: First, compounds like the DNA must be divided exactly, and errors in the duplication and partitioning are the cause of important genetic disorders, such as trisomy 21 (Soltani et al., 2016). Second, components that are present in large numbers may be uniformly segregated stochastically with small relative fluctuations. Third, RNAs, proteins, and organelles are present in numbers low enough that errors in the segregation of individual copies may cause large fluctuations in cell division. Moreover, the presence of large fluctuations or even biases in the partitioning of some compounds can produce a systematic difference in the daughters, which fosters cell differentiation (Shlyakhtina et al., 2019). Furthermore, a cell must restore the correct balance of compounds to ensure that cell progeny does not change too much after several divisions. Given these considerations, it is surprising to notice that as much as partitioning noise appears to take part in several cell division processes, cell populations are characterized by homeostasis that tends to contrast with noise (Rué and Arias, 2015; Taheri-Araghi et al., 2015).

Consequently, we are led to ask ourselves if partitioning noise has an evolutionary advantage and is for this reason selected. Recent observation suggests that asymmetrical partitioning plays an important role in cell-to-cell variability, cell fate determination, cellular aging, and rejuvenation (Huang et al., 2017; Ouellet and Barral, 2012; Raser and O'Shea, 2005). Indeed, (Tobias et al., 2017) found that biased binomial segregation helps bacterial populations to face antibiotic treatments, suggesting partitioning as a drug resistance enhancer. For instance, (Katajisto et al., 2015) have shown that mammalian epithelial stem-like immortalized cells partition mitochondria asymmetrically. Tripathi et al. (2020) use a computational model to show that partitioning is one of the main noise sources behind cancer cell plasticity and they use it to model epithelial-mesenchymal transition (EMT). In their model, partitioning noise increases the heterogeneity of the tumor environment and changes its ability to resist treatment (Tripathi et al., 2020). In this respect, several theoretical and experimental works have shown that in populations of bacteria or cancer cells facing environmental changes, variability increases the probability that some individuals may survive the stress produced by a sudden change in the environment, e.g., the one produced by antibiotics or cancer treatments (Thattai and van Oudenaarden, 2004; Edo and Leibler, 2005; Lu et al., 2007; Castrillo and Oliver, 2011; De Martino et al., 2019; Miotto and Monacelli, 2020). Similarly, mitochondria and endosomes are known to exhibit asymmetrical partitioning in yeast (Boldogh et al., 2001; Rohn et al., 2014; Zhou et al., 2014; Knoblach and Rachubinski, 2015; Chang and Marshall, 2017; Ruan et al., 2017), in asymmetrically dividing cells, like mammalian epithelial stem-like immortalized cells (Dalton and Carroll, 2013), and in symmetrically dividing Jurkat cancer T cells (Peruzzi et al., 2021). This asymmetrical mitochondria partitioning enables cells to protect themselves from aging because it protects the cell progeny from accumulating misfolded proteins engulfed in the mitochondria (Zhou et al., 2014; Katajisto et al., 2015; Ruan et al., 2017; Pernice et al., 2018).

Partitioning errors are not the only source of stochasticity that appear to challenge the homeostasis and morphogenesis of living tissue. There are at least two other sources of randomness (Soltani et al., 2016). First, we have to consider the error and the stochasticity of gene transcription and the rate at which cells reconstitute their pool of sub-components. Second, the last source of error concerns the timing between cells’ division. Given these observations, if observed singularly, a cell is already a complex system. Kauffman (1969) was probably the first to propose that the state of gene expression of a cell is a complex system, postulating that the excitation/inhibition of every single gene is the result of the mutual excitation/inhibition interaction with the state of other genes. These processes may be described by the gene regulatory network (GRN). In this framework, the mutual interaction between genes forms a network. Several works modeled this process with Boolean networks (Kauffman, 1969; Saadatpour and Albert, 2013; Dunn et al., 2014) but there is certainly no reason to restrict ourselves to a model of this kind instead of considering a more descriptive approach that includes the cells’ proteomics and metabolomics (Tosenberger et al., 2017), or another abstract approach that uses Hopfield recurrent neural network (Ryan et al., 2017). In more complex GRNs, there are transcription factors (TFs) that activate and inhibit the transcription of target genes. In turn, target genes produce other TFs or proteins that regulate cell metabolism (Shen-Orr et al., 2002; Mangan and Alon, 2003; Milo et al., 2002). Both logical and continuous methods based on ODEs and information theory were used to model GRNs and capture their stochastic nature (Guy and Shamir, 2008; Tkačik et al., 2008; Tkačik and Bialek, 2016; Monti et al., 2022).

**FIGURE 4:**
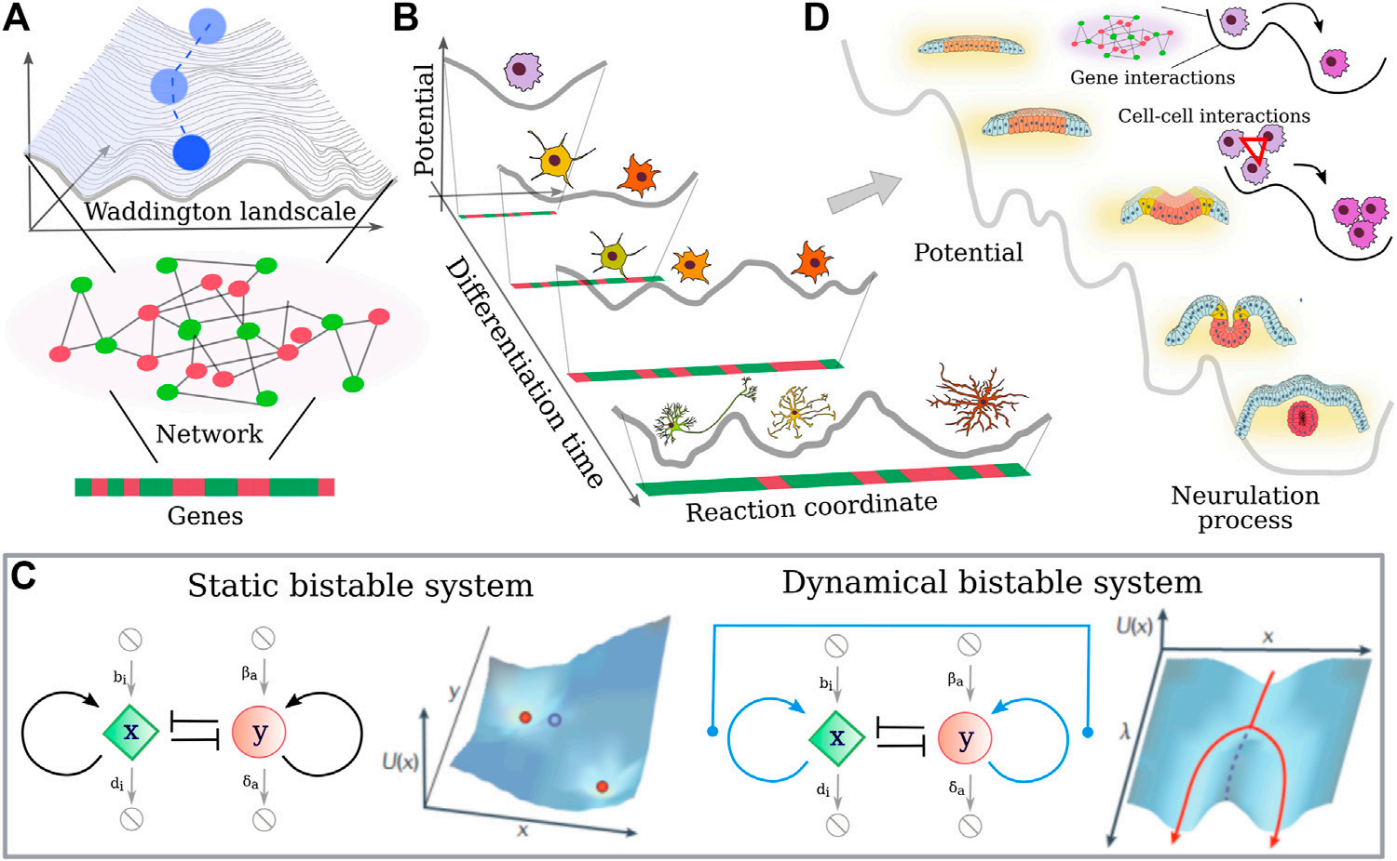
Single cell differentiation vs. collective cell differentiation **(A)** Schematic representation of the Waddington lanscape (Waddington, 1957): a cell, represented as a cobble, starts at the top of a landscape and rolls down via a series of branching points that represent decision events. The shape of the landscape is given by the activity of the underlining cell gene network, where genes can turn on (green) and off (red) during the differentiation time. **(B)** Neural cell differentiation in terms of Waddington landscape. As the cell differentiates, its “differentiation potential” decreases and new valleys can be reached. **(C)** Representation of a bistable molecular circuit in the absence and presence of an external input that allows for tuning of the bistability. The landscape that such small motifs are able to originate suggest a link with the Waddington landscape. In particular, an external parameter dependent on the differentiation time can change the shape of the “potential”. **(D)** Generalization of the Waddington landscape to the case of neurulation, where mechanical interactions between cells cooperate with genetic inputs to facilitate the differentiation process and in turn the neurulation.

Semrau and Oudenaarden, (2015) and Dunn et al., 2014 show how, in response to the state of their gene activation network, pluripotent cells differentiate into progenitor and terminal cells by gradually changing the abundance of their RNA and protein compounds. Given these conditions, researchers started modeling cells as dynamic complex systems in a quasi-stable state (Kauffman, 1969; Saadatpour and Albert, 2013; Dunn et al., 2014; Ryan et al., 2017; Tosenberger et al., 2017). In this mathematical model, cell differentiation and plasticity is the result of perturbations of the cell gene co-expression network that bring the cell from a quasi-stable state into a new one associated with a different cell type (Furusawa and Kaneko, 2012; Miotto et al., 2019). These models may be thought of as a mathematical complex system formulation of the Waddington epigenetic landscape (Waddington, 1957). Indeed, Waddington’s epigenetic landscape is often used as a representation of the relationship between gene network and cell fate (see Figures 4A, B), where fate transitions occur in a stochastic manner and cell signaling modulates the probability of the transition events (Moris et al., 2016).

Note that even if most models assume cell fate decisions to be coordinated through the regulatory interactions between genes, differentiation is not only the effect of transcriptional changes. There are many signals coming from ligand-receptor interactions, cell-cell contacts. These signals converge not only to gene expression, but also epigenetic events and splicing may present additional levels of complexity. Nevertheless, simple network motifs such as the bistable switch (see Figure 4C) can be modeled, and phase planes can be drawn, which identify the stable and metastable points of the system. In this framework, “potential” values for each potential state of the system can be calculated. If a time-dependent parameter of the network is introduced, the dynamic response of the system can be examined. Such parameter changes represent biological situations in which cellular signaling inputs alter the network and affect fate decisions. At critical parameter values, the topology of the system can change, for instance by converting a monostable system with one stable point into a bistable one with two stable and one metastable point. Interestingly, one may speculate that depending on the local topology of such complex landscape and the strength of the perturbation signals, the differentiation process could be also reversed, where differentiated cells types are pushed backward in the hierarchical differentiation process.

Indeed, combining the outcomes of small motifs in more complex interaction networks can give a dynamic landscape highly reminiscent of Waddington’s idea of the epigenetic landscape. Interestingly, the critical parameter can represent chemical stimuli coming from the environment, but so chemical/mechanical interactions occur between the population of cells. Thus a generalization of the Waddington landscape could be proposed by merging the gene interaction-induced differentiation of single cells with the tissue development, where the various phases of the morphogenesis can be reached also thanks to the presence of cell-cell interactions, as depicted in Figure 4D.

## 3 Self-organization and collective behavior

### 3.1 Emergent behavior and active matter

A population composed of several interacting members exhibits an emergent behavior when its members, spontaneously coordinate their behavior producing a global behavior as if the population is become a coherent whole (Prigogine and Nicolis, 1985). In this respect, an emergent collective behavior may evolve even when the single population members neither encode nor predict the population behavior, and for this reason, the collective behavior is solely the result of the population (Vicsek and Zafeiris, 2012). For example, consider the following cases, water evaporation (or any other phase transition) depends on population properties, e.g., temperature, and pressure, which do not apply to a single water molecule, but describe statistically the dynamic interaction among molecules; the foraging of ant-colonies depends on pheromone signaling between few nearby individuals (Dorigo and Birattari, 2011); the coherent movement of bird flocks depends on simple interactions among the neighboring birds (Ballerini et al., 2008). Finally, as described in Turing’s reaction-diffusion model, pattern formation, can be modeled as the result of emergent behavior (Turing, 1952). Cell rearrangements at high densities show features remarkably similar to dynamical heterogeneity in glasses, as shown by Angelini et al. (2010). However, unlike colloidal glasses, cells are self-propelled objects and autonomous motion is a key ingredient that cannot be in general neglected in modeling.

Active matter provides a powerful framework for rationalizing emerging properties in collections of self-propulsive agents interacting in diverse ways (Cates, 2012; Vicsek and Zafeiris, 2012; Marchetti et al., 2013; Cavagna and Giardina, 2014; Bechinger et al., 2016). For instance, signaling interactions promoting local alignment brings to collective polar order, as in the case of the celebrated Vicsek model (Vicsek et al., 1995). Minimal mechanical interactions are very effective for gaining insight into different collective phenomena ranging from bacterial ratchet motors up to activity-driven phase separation (Di Leonardo et al., 2010; Cates and Tailleur, 2015). The current active matter perspective of cell population proposes that, in healthy tissue, cells take a spatial configuration that is the result of a dynamical process. Szabó et al. (2006) discuss a variant of the particle-based Visek model, and show how keratocyte cell motion is characterized by a density-dependent phase transition. As the number of cells per area increases the cells go from a disordered gaseous-like movement to an ordered collective migration. Particle-based models are quite effective in modeling a variety of collective behaviors in dense materials (Paoluzzi et al., 2022a; Paoluzzi et al., 2022b). However, in some situations of practical interest, as in the case of a confluent monolayer where cells are so packed to fill the entire space available, cell shape with its fluctuations is an ingredient that cannot be left away.

Cell shape can be incorporated in different ways as discussed, for instance, in the works of Trepat and Sahai (2018); Camley and Rappel (2017); Alert and Trepat (2020). In the case of Vertex and Voronoi models each cell is represented by a polygon resulting from the Voronoi tessellation of the cell centers. It has been shown in models, and tested against experiments, that the vertex model of biological tissue undergoing jamming and unjamming transitions has a single control parameter which is the typical cell shape (Bi et al., 2014; Bi et al., 2015; Park et al., 2015; Malinverno et al., 2017).

Once cell motion is incorporated, the model develops a phase diagram where tissue solidification is accompanied by a dynamical slowing down as in the case of supercooled liquid (Bi et al., 2016). Alignment interactions drive collective motion not only in the fluid phase, as experimentally observed by Malinverno et al. (2017), but also a peculiar migratory pattern in the solid phase (Giavazzi et al., 2018). Early studies (Henkes et al., 2011) considered alignment interactions that do not depend on cell shape. In (Paoluzzi et al., 2021) some of us explored the impact of continuous feedback between cell displacement and cell motion showing that it deeply impacts the structural properties in confluent monolayers. Even in absence of self-propulsion, the interplay between random motion and shape fluctuations can develop a variety of glassy dynamics (Sussman et al., 2018; Li et al., 2021). Although biological systems are far from equilibrium, altogether these works indicate that collective migration, as well as spatial organization, can be described by the use of an effective energy functional whose minimization allows us to determine the optimal configuration of the system. Moreover, it shows that the fundamental microscopic measurable variables are four, i.e., the cells’ size, shape, major axis, and velocity. Up to now, this research line focuses mostly on cell models in which cells do not differentiate. However, recent studies have pointed out the importance of considering cells of different mechanical properties (Li et al., 2019). This kind of differentiation plays an important role in tumorigenesis and metastasis invasion (Fuhs et al., 2022). Moreover, glassy dynamics is strongly linked with geometrical frustration and, under this perspective, particle-based models that implement a polydisperse mixture of active particles might help in understanding the role of cell-size differentiation in collective rearrangements (Paoluzzi et al., 2022b).

Neural tube formation belongs to a larger class of morphogenetic events called invagination, e.g., *Drosophila* gastrulation, primitive streak (Alt et al., 2017). Several vertex models were proposed to model the evolution of a 2D section, which demonstrated the role of apical constriction and polarized cell shapes (Odell et al., 1981; Rauzi et al., 2015; Štorgel et al., 2016). In this context, (Štorgel et al., 2016) offers a phase diagrame description that allows us to understand how changes in visco/elastic properties change the stationary emergent organization of the tissue. Inoue et al. (2016), instead, introduced a 3D vertex model of neurulation showing that closure is obtained by the interplay of cell elongation in concert with apical constriction with the additional fundamental role of migration of the “deep” cells underlying the neural plate. Furthermore, the finite element models described by Chen and Brodland (2008); Brodland et al. (2010) demonstrate the specific kinds of strains and stress that are necessary to reproduce neurulation. In parallel, some models offered descriptions of neural plate folding as a 2D elastic sheet with implicit apical/basal polarity (Hannezo et al., 2014).

The recent polarity models, described in Nissen et al. (2018); Nielsen et al. (2020) which are strongly connected with Szeliski and Tonnesen (1992), are able to capture the coordinated role of apical/basal polarity alignment and mechanical interaction. These models effectively balance between realistic detail and synthesis in modeling 3D neural tube morphogenesis. We refer the reader to the review of Wyczalkowski et al. (2012) for a further detailed account of 3D model of neurulation. Unfortunately, these models do not try to understand the collective decision-making process that involves simultaneously single-cell differentiation and cell shape and strain control. Without the collective coordination of these processes neurulation main not correctly terminate. This is particularly evident in neural rosette formation because not all the cells in the culture are able to effectively self-coordinate and form neural rosette patterns and some of the cells that do not take part in the formation of rosette patterns are cells that systematically fail differentiation.

Just a few system biology approaches try to integrate gene expression network models with spatial organization models (Tosenberger et al., 2017). These models use a very sophisticated and detailed model that combines the genomic and proteomic networks, and for this reason, it is very descriptive but it is not synthetic in the characterization of the fundamental nature of the interaction between cells and does not describe the mechanical and elastic interaction among the cells. Contrarily, a synthetic model of cell type determination should not focus on the full cells’ gene expression, but on the relational mechanism that forces interacting cells to take the same fate or to push each other onto a different path.

### 3.2 Emergent behavior and phase transitions

**FIGURE 5:**
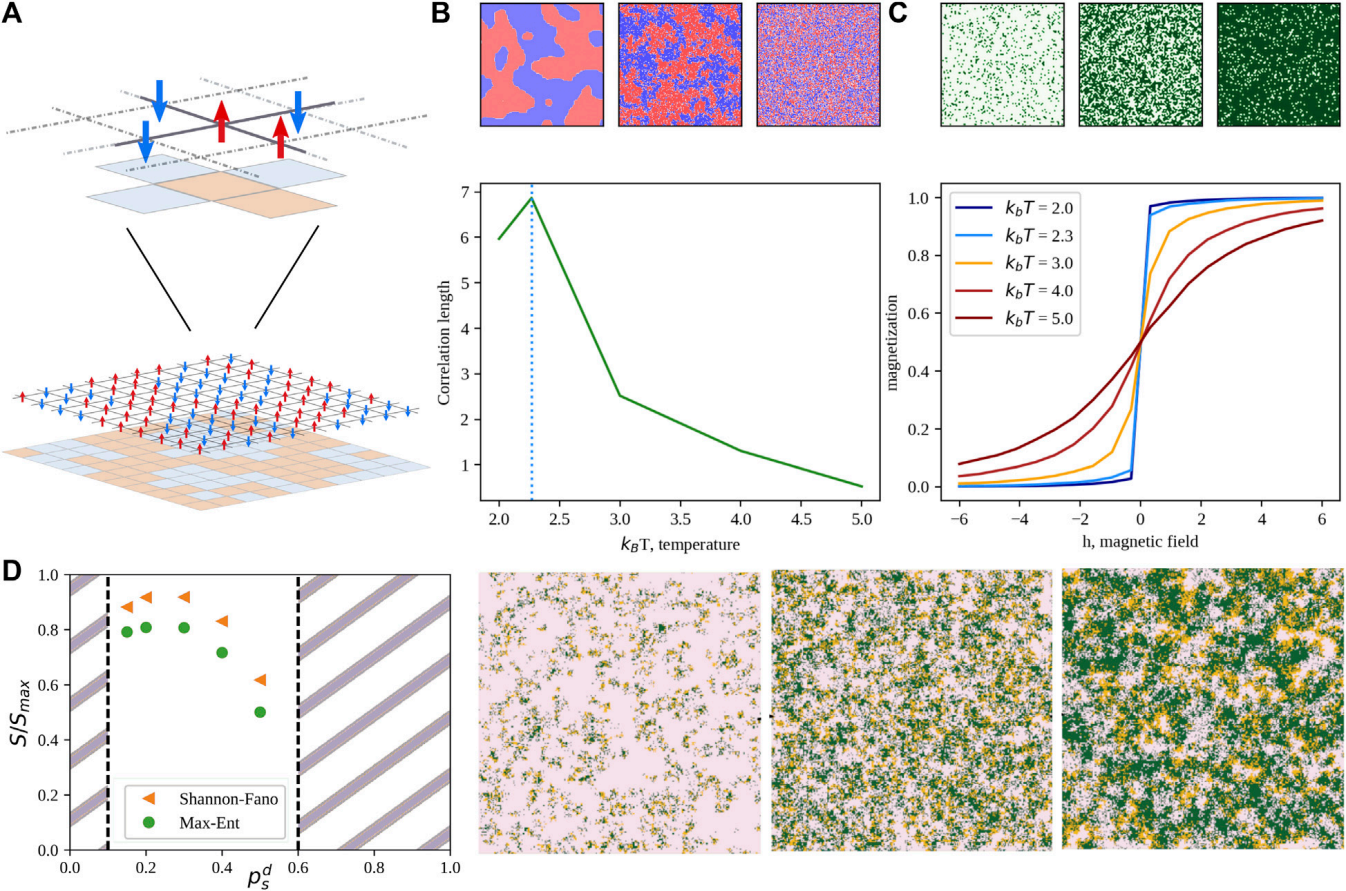
Phase transitions and the generalized Ising model. **(A)** Schematic representation of the two-state Ising model on a 2D lattice. Red arrows represent the “up” state, while blue ones the “down” orientation. **(B)** Correlation length of the “up” state as a function of the scaled temperature. The dotted vertical line marks the critical temperature. Colormaps above the plot depict, from left to right, a sub-critical, critical, and over-critical configuration, respectively. **(C)** Magnetization of the “up” state as a function on the external field, *h*. Curve colors range from blue to dark red as the scaled temperature increases. Colormaps above the plot depict, from left to right, configurations obtained with *h* = −5, 0, and 5, respectively. **(D)** Example of an application of a generalized Ising model to the description of the non-equilibrium steady state of a prey-predator model (Miotto and Monacelli, 2018; Miotto and Monacelli, 2021). Normalized entropy as a function of the predator motility obtained neglecting interactions (Shannon-Fano) or considering near-neighbor correlations (Max-Ent). Snapshots are taken from the system steady-state distribution while tuning the predator mobility from 0.47 (left) to 0.90 (right). Preys are depicted in pink, predators in orange and empty sites are colored in green.

As an example, of an emergent collective behavior, consider, for example, a complex system characterized by two states, *D* and *O*, each state characterized by a certain degree of order or organization, such that *O* is the more ordered state and *D* is the less ordered state. In statistical physics, a phase transition is a transition from the system state *D* to the state *O*. The order of the system is measured through the order parameter, which is a mathematically defined quantity, that quantifies the degree of order of the system. The order parameter is derived from the observation and characterization of fundamental variables describing the emergent collective behavior (Parisi, 1988). Such that the less-ordered state *D* will have a lower value of the order parameter if compared to the more-ordered state *O*. In general, in the transition from a less ordered phase to a more ordered state, the system losses the broadness and homogeneity of behaviors adopted by the single population members, and is forced to select an ordered state in which all members behave more coherently. Thus, an order parameter is usually a statistical measure of the observed behaviors inside the population which describes the broadness of homogeneity in the population. In statistical physics, the Ising model and its various generalization has been developed to account for such processes in magnetic materials. Indeed, one can consider a system composed of elements that assume two different states. In the original model, those elements were magnets either oriented in the ‘up’ state or in the “down” one, but in subsequent generalizations, those elements were as different as cells and amino acids of protein sequences. The Ising model describes the probability of finding a system of those magnets in certain orientations (see Figure 5A), assuming that such probability depends on two parameters, i.e., an external field (*h*) that tends to orient the magnets in a certain direction and a local interaction term (*J*) that link the orientation of each magnet with those of its surrounding magnets. In mathematical terms, the probability of the Ising model is

$$P\left(\left\{\sigma \right\}\right)=\frac{\exp\left(-\beta H\right)}{Z}\;\text{and}\;H=h\underset{i}{\sum }\sigma_{i}+J\underset{i}{\sum }\underset{j,|i-j|=1}{\sum }\sigma_{i}\sigma_{j}$$ (2)

where *σ* is the magnets orientation, by convention represented as ± 1; *β* is the inverse of the temperature and *Z* a normalization factor.

Such a model exhibits peculiar features upon varying the temperature. In fact, if one looks at a typical equilibrium configuration at high temperature (top-right panel in Figure 5B), we found a noisy configuration, with up and down cells randomly distributed in space. Lowering the temperature, clusters of cells with the same orientation start appearing. Interestingly, in finite-sized systems, the average dimension of such clusters has a maximum for a certain critical temperature. Such temperature marks a phase transition of the second order between ordered and disordered phases. Varying the external field parameter, *h* instead modifies the magnetization of the system, as one can see from Figure 5C. Besides its relevance for solid state physics, the Ising model is playing a main role in the area of complex systems as the Hamiltonian (i.e., the energy function) that fully characterize the general Ising or Potts model is the maximum entropy solution for the probability distribution of a system for which densities and correlations are measurable (Miotto and Monacelli, 2021).

Over the past decade, many works employed MaxEnt to analyze different biological problems, ranging from the study of neural populations to the determination of macromolecular structures, to the inference of regulatory networks, and collective behavior in large animal groups (Schneidman et al., 2006; Martin et al., 2009; Cavagna and Giardina, 2014; Santolini et al., 2014). In addition, also the behavior of prey-predator systems has been shown to be well reproduced by a three-state Potts Hamiltonian (Miotto and Monacelli, 2018), where predator motility tuned out to be an order parameter for such systems (see Figure 5D).

Along this line, if one considers how epithelial cells align their velocity, we find that as predicted in Szabó et al. particle-based Visek model (Szabó et al., 2006), there are two main regimes. Cells are found in a disordered state *D*, at low density and seldom touch each other, where they move randomly and select their direction homogeneously at random. Contrarily, in an ordered state *O*, cells are found at high density and form a confluent layer, in this condition cells move all together selecting collectively an emergent direction.

In this case, (Szabó et al., 2006), the order parameter is the alignment which is defined as the average of the cells’ velocity vector. This is a good order parameter because it is a statistical measure that characterizes the level at which the population moves in an orderly direction. In state *D*, cells move in all directions homogeneously, thus for the central theorem, the alignment for a group of cells tends to zero as the size of the group increases. In state *O*, cells move in a selected direction with a fixed velocity, thus the alignment is equal to the displacement vector of the cell population. Averages of characteristic measurable quantities of the system are often good order parameters because tend to zero when the measurable quantity gives noisy values homogeneously spread over all possible outcomes, and tend to a value greater than zero when the observed population members’ behaviors converge to a certain degree of uniformity or organization.

**FIGURE 6:**
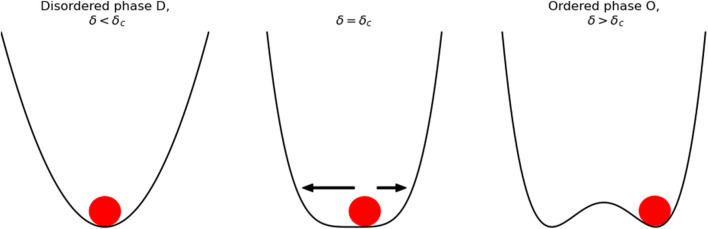
Symmetry breaking diagram. For *δ* > *δ*_*c*_ the ball falls in a single valley where the states are disordered and point homogeneously in all directions. For *δ* < *δ*_*c*_ the ball has to fall in one of two valleys selecting a specific order, the selection of one of these valleys is called symmetry breaking.

Phase transitions are observed in several physical and biological systems and share several features. One of the main characteristics of a phase transition is an abrupt change of the order parameter, in response to a gradual modification of one of the fundamental system parameters, such as density, or pressure. In Szabó et al. (2006), the phase transition is the result of the different cell density levels. Another general characteristic is that as the order parameter increases the system is forced to a specific order state among the different available ordered states. In Figure 6, for *δ* < *δ*_*c*_ the cell population is found in a single minimum which represents the disordered state, for *δ* > *δ*_*c*_ the cell population is forced to choose an ordered configuration among the possible configurations available, Figure 6 presents two minima one for each possible population configuration. At a macroscopic level, this process determines the ability of a system composed of many members to take a collective decision, acting as an individual which determines its behavior. In the cell sheet case, (Szabó et al., 2006), the cells select a single direction of motion among several, which is then followed by all cells. The emergent selection of a uniform configuration, among several possible configurations is often referred to as spontaneous symmetry breaking.

## 4 Discussion

In the previous sections, we explored the main theoretical frameworks that have evolved to explain morphogenesis. Now, we will focus on what is still missing (Section 4.1) and what may help making further progresses Section 4.2), possibly viewing morphogenesis as a phase transition process (see Section 4.3).

### 4.1 Current research gap

We start discussing the explanatory power and the limits of the cited frameworks. In particular, we began with the molecular biology framework, and then we gradually explored the diffusion models and cell sorting explanatory frameworks. Finally, we entered the current model connected to soft matter, collective behavior, and finite element models.

The molecular biology description of cell differentiation is very effective at capturing the transcriptional and translational mechanisms that regulate stem cell function (Saba et al., 2021). More precisely, molecular biology models can account for the role of genes in activating differentiation at the proper developmental time point. Gene activity is characterized by signaling pathways and transcriptional regulators that work together to form a dynamically complex system capable of responding to external stimuli and active lineage fate determination (Rauch and Mandrup, 2021). Unfortunately, molecular biology models are limited by their ability to account for the physical processes required for self-organization in morphogenesis because they do not incorporate the mechanical and spacial constraints that guide tissue development (Collinet and Lecuit, 2021). In practice, these models do not give us an explanation of the rosette pattern formation, but only an explanation of the cell differentiation in neural progenitors inside the rosette. From another point of view, diffusion models are very effective in producing a large variety of patterns but their experimental validation has been limited. Furthermore, while diffusion models help us understand the formation of stable morphogen patterns they do not help us understand the processes of cell migration and mechanical sensing that have been observed in morphogenesis. In analogy, cell sorting allows us to understand migration and the organization of specific cell types in different tissue but it lack the ability to explain the differentiation changes that are present at the single-cell level. Thus, these models do not allow understanding the causal relationship between cell fate determination and tissue pattern development. Considering the more current counterpart, finite-element and agent-based models of morphogenesis allow us to understand the mechanical conditions required for pattern formation, but do not explain how these mechanical conditions are determined through supposedly a self-organizing system based on the interaction between mechanical signaling and the cell transcriptional and translational mechanisms. Collective behavior and active matter models give us a powerful framework to understand self-organization but at the moment there have been limited applications for neurulation and neural rosette formation, and these applications disregarded the relation between cell differentiation, and pattern formation.

### 4.2 Potential developments in the field

In this review, we evaluated the evidence showing how neural rosettes are a good testing ground for studying morphogenesis. 3D simulation models are paramount to understanding the fundamental mechanical interactions among the cells required for correct neural tube closure. Unfortunately, these models are computationally expensive, as even a single run may require powerful computers and extensive computational time. Consequently, it is hard to understand the global mechanical properties of the system that are required to study the stability under perturbations, and the zoology of potential stable and meta-stable states that may lead the system out of its proper healthy state into dysfunction or disease (Collinet and Lecuit, 2021).

For this reason, a simplified model, potentially a 2D model would be very helpful for the study of the full global analysis that is required to understand the landscape that characterizes the evolution of the neural tube. To this aim, neural rosettes morphogenesis is the ideal biological model because it incorporates the fundamental neural tube morphogenetic transformations, and it is, at the same time, realistic to assume that it can be modeled with a multi-agent system that incorporates self-organization and collective single-cell fate determination. Furthermore, it incorporates the fundamental neural tube morphogenetic transformations ranging from specific molecular biology processes to similar mechanical and shape modifications. Finally, it is naturally a 2D model.

A computational model of neural rosette morphogenesis would allow us to develop phase diagrams and to understand the system parameters that allow the system to self-organize transiting from a disordered state to a more ordered state. This model will guide the design of experimental conditions that can demonstrate the validity of the models first on 2D neural rosettes and then in 3D neural tube formation experiments.

### 4.3 Tissue development as a sequence of phase transitions

Regardless of the specific model used to describe a cell, considering a cell as a complex system, we expect that given a certain external environment, the cell will tend to be in a specific macroscopic state or phase (Furusawa and Kaneko, 2012). Furthermore, given that the cell is a complex system, we find that the cell state is characterized by a multidimensional vector and that when a cell is found in a certain differentiation state this multidimensional vector will travel along a certain attractor cycle, such that for each cell type there will correspond a specific dynamic attractor in the gene expression space. The cell attractor will characterize a trajectory that is stable in time and robust to transcription errors and other sources of noise. Nevertheless, this attractor may be perturbed when the cell is exposed to external signals such as chemical signals (e.g., soluble factors or membrane junctions), or mechanical interactions. These external signals mediate the interaction between the cells and the surrounding environment. Thus, since a cell population is composed of several cells each characterized by a certain single-cell attractor state, which interacts through mechanical and chemical signals, we can consider the tissue formed by this population as a complex system composed of smaller complex systems. Thus, the formation of tissue requires the coordination of the single-cell transcriptomic states. Specifically, in the case of spatially organized tissue, as for the neural tube, it requires the cells’ transcriptomic states to be coordinated according to the mutual position among the cells, and to the global positional information of the organism (Gregor et al., 2007). Thus, in this prospective, tissue morphogenesis may emerge from the interplay between two interacting dynamical systems characterized by different scales: the genetic expression dynamics, and the tissue self-organization dynamics. Given this perspective, we find that development is usually characterized either as a sequence of differentiation processes at the single-cell level or as morphogenetic events that generate different tissue, this paper suggests that these two frameworks describe the same process at two different scales, and that these scales can be coherently studied as a sequence of self-organization events that can be described as phase transitions, using the methods developed in statistical physics.

## 5 Conclusion

Understanding neural tube formation is of fundamental important because errors in neural tube formation are the cause of major developmental disorders. In this contest, neural rosettes can be used as a 2D model to study the 3D self-organization of the neurolation process. Here, we discussed how neural rosette cannot be separately described either as a cell differentiation process, or as a patterning process, but a complex system perspective that merges these different perspectives is required. Cells that form rosettes change their gene network activation state as shown by the expression of PAX6, ZO-1, and N-cadherin, while cells outside the rosettes do not always express such markers. Although we have several information about gene activation and differentiation, and we are able to model several aspects of pattern formation, we still lack a coherent theoretical framework to study these two aspects together in a single multi-scale phenomenon. We argue that active matter and collective behavior models may be the best candidates for this task, as the physics of phase transition may allows us to connect our understanding of cell differentiation with the emergent self-organization of tissue. In fact, active matter and collective behavior give us a way to connect the stochastic decision process at the single-cell level with the global tissue-level mechanical transformations. To do this, we should develop a model that captures the stochasticity at the single-cell level, together with a sufficient description of the chemical signal and mechanical cues, necessary to describe the single-cell fate determination. The single-cell fate determination must then be linked to the consequent single-cell stress and strain that drive the mechanical transitions into the emergent pattern (Hannezo et al., 2014). In this framework, one of the questions would whether it is cell fate determination that guides single-cell mechanical changes or if single-cell mechanical changes guide cell fate. Possibly, it may be a feedback loop. These questions can be extensively explored in rosette formation because they can be naturally modeled in 2D without making limiting assumptions, and because modeling hypothesis and observation can be readily tested on *in vitro* experiments. In Eric F. Wieschaus’s words, we would like to understand “[…] how genetic activity is translated into the physical properties that govern mechanics, or govern movement or change in shape. […] We want to understand it the way an engineer understands how you build a bridge, a self-building bridge.” (Kavli Institute for Theoretical Physics, 2018). The fact that tissue emerges from the self-organization of cell population has several inherent benefits, the system is robust to small single-cell errors and genetic mutations, furthermore, the genetic code that encodes the information required for the production of the morphogenetic events is not required to specify the complete description of the dynamic evolution of each cell in the population, as an engineer would need to plan the construction of a bridge (rephrasing Weischaus words), but it has to encode an effective Lagrangian that guides the single cell decision making. In such a way that it would allow the cell to adapt to the surrounding environment and self-organize collectively in the desired structure.
